# The S40 residue in HIV-1 Gag p6 impacts local and distal budding determinants, revealing additional late domain activities

**DOI:** 10.1186/1742-4690-10-143

**Published:** 2013-11-21

**Authors:** Susan M Watanabe, Min-Huei Chen, Mahfuz Khan, Lorna Ehrlich, Kimdar Sherefa Kemal, Barbara Weiser, Binshan Shi, Chaoping Chen, Michael Powell, Kathryn Anastos, Harold Burger, Carol A Carter

**Affiliations:** 1 Department of Molecular Genetics & Microbiology, Stony Brook University, Life Sciences Bldg. Rm 248, Stony Brook, NY 11794-5222, USA; 2 Department of Microbiology & Immunology, Morehouse School of Medicine, Atlanta, GA 30310, USA; 3 New York State Department of Health, Wadsworth Center, Albany, NY 12201, USA; 4 Colorado State University, Fort Collins, CO 80523, USA; 5 Montefiore Medical Center, Bronx, NY 10467, USA; 6 Albert Einstein College of Medicine, Bronx, NY 10461, USA; 7 Current address: Albert Einstein College of Medicine, Bronx, NY 10461, USA; 8 Current address: University of California Davis School of Medicine and Sacramento VA Medical Center, Sacramento, CA 95817, USA; 9 Current address: Albany College of Pharmacy and Health Sciences, Albany, NY 12208, USA

**Keywords:** HIV-1, Alix, Tsg101, Nedd4, CA NTD, Viral particle maturation, Protease, Viral budding

## Abstract

**Background:**

HIV-1 budding is directed primarily by two motifs in Gag p6 designated as late domain-1 and −2 that recruit ESCRT machinery by binding Tsg101 and Alix*,* respectively, and by poorly characterized determinants in the capsid (CA) domain. Here, we report that a conserved Gag p6 residue, S40, impacts budding mediated by all of these determinants.

**Results:**

Whereas budding normally results in formation of single spherical particles ~100 nm in diameter and containing a characteristic electron-dense conical core, the substitution of Phe for S40, a change that does not alter the amino acids encoded in the overlapping pol reading frame, resulted in defective CA-SP1 cleavage, formation of strings of tethered particles or filopodia-like membrane protrusions containing Gag, and diminished infectious particle formation. The S40F-mediated release defects were exacerbated when the viral-encoded protease (PR) was inactivated or when L domain-1 function was disrupted or when budding was almost completely obliterated by the disruption of both L domain-1 and −2. S40F mutation also resulted in stronger Gag-Alix interaction, as detected by yeast 2-hybrid assay. Reducing Alix binding by mutational disruption of contact residues restored single particle release, implicating the perturbed Gag-Alix interaction in the aberrant budding events. Interestingly, introduction of S40F partially rescued the negative effects on budding of CA NTD mutations EE75,76AA and P99A, which both prevent membrane curvature and therefore block budding at an early stage.

**Conclusions:**

The results indicate that the S40 residue is a novel determinant of HIV-1 egress that is most likely involved in regulation of a critical assembly event required for budding in the Tsg101-, Alix-, Nedd4- and CA N-terminal domain affected pathways.

## Background

Gag, the structural precursor polyprotein encoded by all retroviruses, is necessary and sufficient for assembly of the immature viral particle [1]. Within the Gag polyprotein, the p6 region harbors important determinants of viral budding (reviewed in [2-4]). A now apparent aspect of Human Immunodeficiency Virus type 1 (HIV-1) replication is the existence of multiple independent pathways for exit of progeny virus particles from the host cell. These pathways involve direct interaction between members of or adapters of cellular ESCRT complexes and budding determinants in the virus encoded structural precursor polyprotein, Gag. Typically, budding of wild-type (WT) Gag virus-like particles (VLPs) or of authentic virus particles requires the binding of Tsg101, a component of ESCRT-I, to the P_7_TAP motif in the C-terminal p6 region of Gag (ESCRT = endosomal sorting complex required for transport; ([5-7]; reviewed in [8-10]). The motif is designated as Late (L) domain-1. When Tsg101-PTAP interaction is disrupted, *e.g*., in the Gag mutant (P7L) where Leu has been substituted for Pro7, budding becomes dependent on L domain-2 in p6 (LY_36_PX_2_S_40_L; where X are highly, but not absolutely, conserved amino acids [11-13]. This second budding pathway requires Alix interaction with Gag through both p6 and determinants in nucleocapsid (NC) [14]. Alix is an ESCRT adapter protein which, upon oligomerization, links Gag to CHMP4 subunits in ESCRT-III. Alix-mediated egress also requires Nedd4 [15]. In the absence of a functional L domain-1 and −2, viral particles can still continue to use the cellular machinery to bud through the plasma membrane through an E3 ubiquitin ligase-dependent pathway. A Nedd4 isoform, Nedd4-2s (Nedd4L-ΔC2) can promote virus release when L domain-1 is disrupted, whether or not L domain-2 is intact [16,17]. The Nedd4 family of E3 ubiquitin ligases bind to PPXY (designated as PY) motifs in several of the retroviral Gag proteins and ubiquitinate these proteins [18-20]. Interestingly, although HIV-1 Gag does not have a PY motif, (*i*), it is known to be Ub-modified [21,22]; (*ii*), viral budding is impacted by translational fusion of Ub to Gag [23-26]; (*iii*), viral budding and infectivity have been shown to be affected by changes in the levels of free cellular Ub [23]; and (*iv*), Tsg101 has been reported to associate with Nedd4 [16,27]. Thus, it is likely that Nedd4 plays a role in HIV-1 egress.

L domain-1 is intact in all HIV-1 variants isolated to date (Los Alamos National Laboratory (LANL) HIV database). For this reason, L domain-2 is thought to have a secondary redundant function versus L domain-1. Particularly intriguing is the high conservation of residue 40 in L Domain-2 given that, based on structural studies [28-31], it is not a residue that contacts Alix. Since p6 in the *gag*-ORF overlaps with the transframe region (p6*) in the *pol*-ORF, residue 40 is Ser (S) in the context of the Gag precursor and Phe (F) in the context of the Gag-Pol precursor where it occupies the P1 position of the p6*/PR cleavage site. Notwithstanding, it was recently reported that the substitution of F for S at residue 40, a mutation that does not alter the sequence of the overlapping pol frame where the viral PR is encoded, results in a loss of infectivity due to inefficient cleavage at the capsid-spacer p1 (CA-SP1) junction [32]. Cleavage at this site in the Gag precursor is required for the morphogenetic rearrangements that produce the infectious particle ([33,34]; reviewed in [35-37]). This observation led the authors to conclude that the p6 region may function in viral maturation independently of its role in L domain-mediated release.

In this report, we confirm that the S40F mutation results in defects in particle maturation. Furthermore, we show that the mutation altered the budding mediated by determinants in both the p6 and the CA domains of Gag. These studies suggest that the S40 residue in p6 is an important determinant of a viral particle release step that regulates an event common to the many budding pathways used by the virus.

## Results

### Mutation of S40 to Phe (S40F), which does not alter the amino acid sequence in the overlapping pol reading frame, nevertheless inhibits CA maturation, alters budding, and reduces viral infectivity

**Figure 1:**
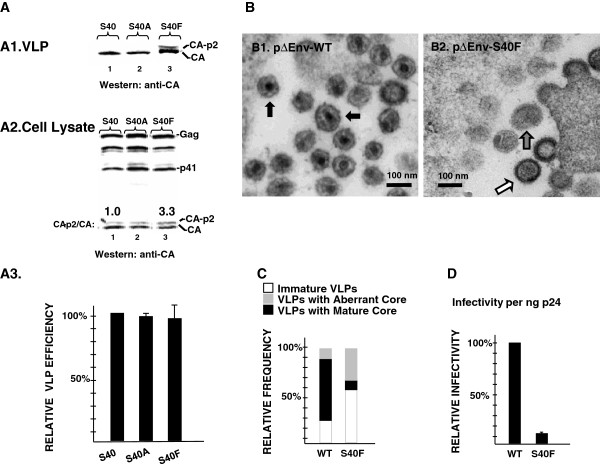
**Mutation of S40 to Phe, which does not alter the amino acid sequence in the overlapping pol reading frame, nevertheless inhibits CA maturation, alters budding, and reduces viral infectivity.** Panels **A1-A3**, Western blot analysis of pNL4-3ΔEnv WT, S40A and S40F constructs which encode active protease. VLPs and cell lysates from COS-1 cells transfected with pNL4-3ΔEnv-WT (*lane 1*), pNL4-3ΔEnv-S40A (*lane 2*) or pNL4-3ΔEnv-S40F (*lane 3*) were analyzed by Western blotting. The values in the figure indicate the ratio of p25/p24 normalized to wild type. The relative VLP efficiencies [VLP/(VLP + Gag from cell lysate)] were determined as described in Methods. Panel **B**, Electron microscopy of particles associated with cells transfected with pNL4-3ΔEnv-WT **(panel B1)**, or S40F **(panel B2)**. Immature particles (*open arrows*), particles with aberrant cores (*gray arrows*), and mature particles with conical cones (*solid arrows*) were released from pNL4-3ΔEnv-transfected cells. Panel **C**, *Quantitative analysis*. 25 WT or 100 S40F VLPs (n =2) were counted and the frequency of each type of particle was determined. Panel **D**, Viral particle infectivity, determined by MAGI assay, done in triplicate.

To confirm the previously observed processing defect of S40F, we engineered the mutation into pNL4-3-ΔEnv and examined Gag processing products by Western analysis. The pNL4-3ΔEnv construct encodes an active PR and is capable of producing mature, noninfectious particles [38]. Consistent with a previous finding [32], we observed that S40F interfered with CA-SP1 to CA processing, mature particle formation, and viral infectivity when engineered into the pNL4-3 background. As expected, in the VLPs detected in the media of COS-1 cells transfected with pNL4-3ΔEnv-WT, proteolytic processing generated almost exclusively the mature CA p24 protein (Figure 1, *panel A*, *lane 1*). In contrast to WT and consistent with the results of the previous study [32], the particles released from cells transfected with S40F contained significant levels of CA-SP1 (p25), indicating that processing at the CA-SP1 junction was defective (*panel A*, *lane 3*). The levels of accumulation of Gag and Gag-related products inside the cells appeared comparable although the processing of CA-SP1 to CA was consistently reduced in cells expressing the S40F mutation (*compare lanes 1 and 3*). Unexpectedly, the mutation of S40 to A, a change that alters the amino acid sequence at the p6*-PR cleavage site in the overlapping pol frame, resulted in WT levels of CA-SP1 to CA processing, as assessed by Western blotting: VLPs released from cells expressing S40A exhibited no defect in CA-SP1 to CA processing (Figure 1, *panel A*, *lane 2*). Thus, the impact of S40F is surprising. The current study focuses on S40F.

S40F did not significantly affect the apparent viral budding efficiency [CA-related signal intensity in VLP/(VLP + cell lysate)], as judged by Western analysis (Figure 1, *panel A3*). However, S40F produced a higher percentage of immature or aberrant mature particles, as indicated by electron microscopy (*panel B*). As shown in *panel C* pNL4-3ΔEnv-WT produced three types of particles: (*i*) immature VLPs, which have no internal core; (*ii*) VLPs with aberrant internal core structures; and, predominantly, (*iii*) VLPs that exhibited the cone-shaped internal core structure characteristic of the mature, infectious WT virus (*solid arrow in panel B*, *left*). The same three types of particles were found when cells were transfected with pNL4-3ΔEnvS40F (*panel B*, *right*) except that the S40F mutant produced significantly fewer particles with conical cores and relatively more immature VLPs (*open arrow*) and VLPs with aberrant cores (*gray arrow*). Approximately 50%, and 13% of the particles produced by pNL4-3 ΔEnv-WT, and -S40F, *respectively*, possessed mature core structures (*panel C*). The results indicate that S40F produced mature particles at a lower frequency than the WT and are consistent with the observation that the S40F mutant was more defective than the WT in CA maturation. To evaluate viral particle infectivity, cells were co-transfected with plasmids pNL4-3ΔEnv-WT, or -S40F and pIIIB-Env-3-1 and the specific infectivity per ng p24 in the cleared cell media was determined in single-round assays using MAGI cells [39]. In agreement with a previous study [32], the substitution of F for S40 reduced viral infectivity 8- to 10- fold compared to particles produced by the WT (*panel D*), (n = 6). The results indicate that the S40F mutation impairs CA-SP1 processing, production of mature particles with conical cores, and, thereby formation of infectious virus without affecting the viral-encoded PR. S40F is therefore impacting a Gag-related assembly function.

### The defect resulting from S40F mutation becomes more apparent when PR is inactive

**Figure 2:**
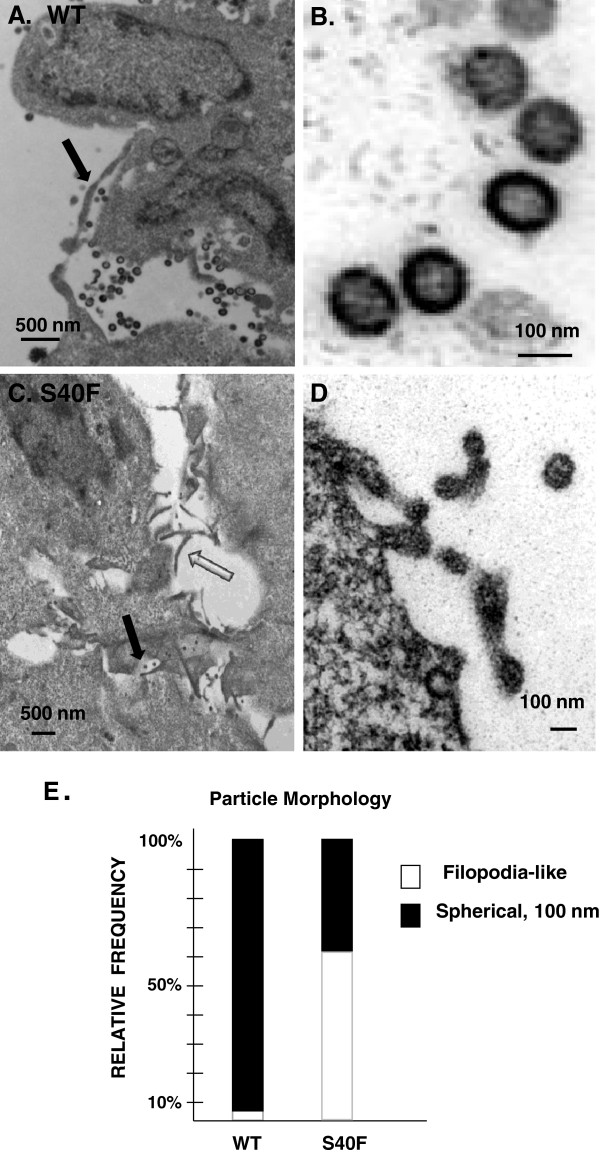
**The budding defect resulting from S40F mutation becomes more apparent when PR is inactive.** COS-1 cells were transfected with plasmids expressing HA-tagged HIV-1 Gag*-*WT **(panels A and B)** or Gag-S40F **(panels C and D)**. Cells were prepared for examination by electron microscopy as described in Methods. Panels **A** and **C***, solid arrows*, immature particles; *open arrows*, extended or tethered particles. Bars in panels **A** and **C** measure 500 nm; bars in panel **B** and **D** measure 100 nm. The relative frequencies of typical spherical particles and filopodia-like particles are summarized **(panel E)**.

Compared to WT, the VLPs made by pΔEnv-S40F appeared to remain more cell-associated (*c.f*., *panel B*). This subtle change, in addition to the observation that they were predominantly immature in morphology despite the presence of mature p24, suggested that they had not been fully released from the cell and were thereby blocked at the CA maturation cleavage which is a very late event. To determine whether the mutation affected viral assembly prior to Gag processing, we used thin section electron microscopy to examine the particles produced by pHIV-1-*gag*-*HA* in which the PR is absent. It is well-established that, all by itself, WT Gag is sufficient for assembly and release of immature VLPs [1]; (Figure 2, *panels A and B*). Immature particles were also detected in the culture transfected with DNA encoding S_40_F-Gag. However, in contrast to the single spherical VLPs which the WT produced almost exclusively, we observed that the majority of particles produced by S40F consisted either of spherical structures that remained tethered to each other in short chains or long (0.5 to 1.0 μm) membrane extensions that resembled filopodia (*panels C and D*). The relative frequency of particles by morphology is summarized in *panel E*.

### Disrupting L domain-1 increases the effect of the S40F mutation on budding

**Figure 3:**
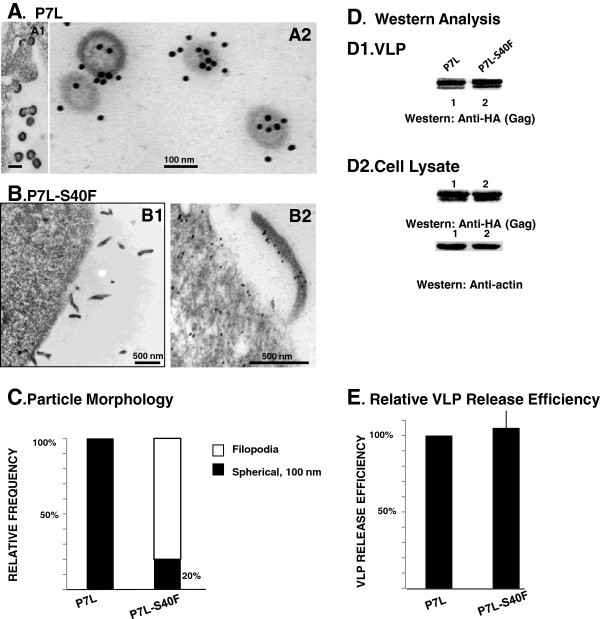
**Disrupting L domain-1 increases the effect of the S40F mutation on budding.** COS-1 cells were transfected with plasmids expressing HA-tagged HIV-1 Gag-P7L **(panels A1 and A2)** or P7L-S40F **(panels B1 and B2)** and prepared for examination by electron microscopy as described in Methods. Panels **A2**, and **B2**: Samples were labeled with primary rabbit anti-CA antibody that was tagged with 15 nm gold-conjugated mouse anti-rabbit secondary antibody (bars in *panels A1 and A2* measure 100 nm; bars in panels **B1** and **B2** measure 500 nm). Panel **C**, *Quantitative analysis of particle morphology*. 200 gold-tagged VLPs were counted and the frequency of spherical *versus* extended structures were determined (n =2). Panel **D**, Western Analysis. VLPs and cell lysates from the COS-1 cells transfected with HA-tagged HIV-1 Gag-P7L (*lane 1*), P7L-S40F (*lane 2*) were analyzed by Western blotting and gag related proteins identified by monoclonal HA antibody. Panel **E**, the relative VLP release efficiencies [VLP/(VLP + Gag from cell lysate)] were determined as described in Methods.

To determine whether the S40F mutation affected viral assembly prior to particle budding, the L domain-1 (P_7_TAP) was mutated to inhibit virus release. Cells were transfected with P_7_L-Gag or P_7_L-S_40_F-Gag in which the P_7_L mutation eliminates Tsg101 binding and results in inefficient bud scission. As can be seen in Figure 3, the cells transfected with P_7_L-S_40_F-Gag exhibited few of the typical virus-like particles and instead were associated with elongated particles and filopodia-like structures (Figure 3, *panel B*). In contrast, as expected, the surface of cells transfected with the parental P_7_L-Gag accumulated single bud evaginations and tethered lollipop-like structures (*panel A*). To confirm that Gag was associated with the membrane extensions, we examined the structures by immunoelectron microscopy. Cells were transfected, thin-sectioned for electron microscopy, and then labeled with primary rabbit anti-CA antibody that was tagged with 15 nm gold-conjugated mouse anti-rabbit secondary antibody. The VLPs produced by P_7_L-Gag were labeled with the gold-tagged antibody probe (*panel A2*), as expected. In addition, cells in cultures transfected with P7L-S_40_F possessed membrane extensions labeled with the gold-tagged probe (*panel B2*) indicating that Gag was associated. In some cases, the extensions contacted neighboring cells, which is reminiscent of structures reported for HIV-infected dendritic cells where viral egress is coupled to virus-induced filopodia formation [40] A quantitative analysis (*panel C*) in which ~200 gold-tagged structures were examined in each culture indicated that the P_7_L-S_40_F mutant produced the filopodia-like structures to a significantly greater extent than the P7L parent. VLP release efficiency was not affected for the P_7_L-S_40_F mutant in comparison to P_7_L (*panel D*, *Western blot analysis*; *panel E*, *VLP release efficiency*). That Gag accumulated in aberrant plasma membrane extensions rather than the characteristic spherical structures under these conditions (*i.e*., disrupted PTAP motif) as well as manifesting aberrant egress under conditions when PTAP was intact suggested that S40 affected a stage in the virus production pathway closely linked to budding whether release was directed by L domain-1 or L domain-2.

### The S40F mutation increases Alix binding to Gag in the yeast 2-hybrid assay

**Figure 4:**
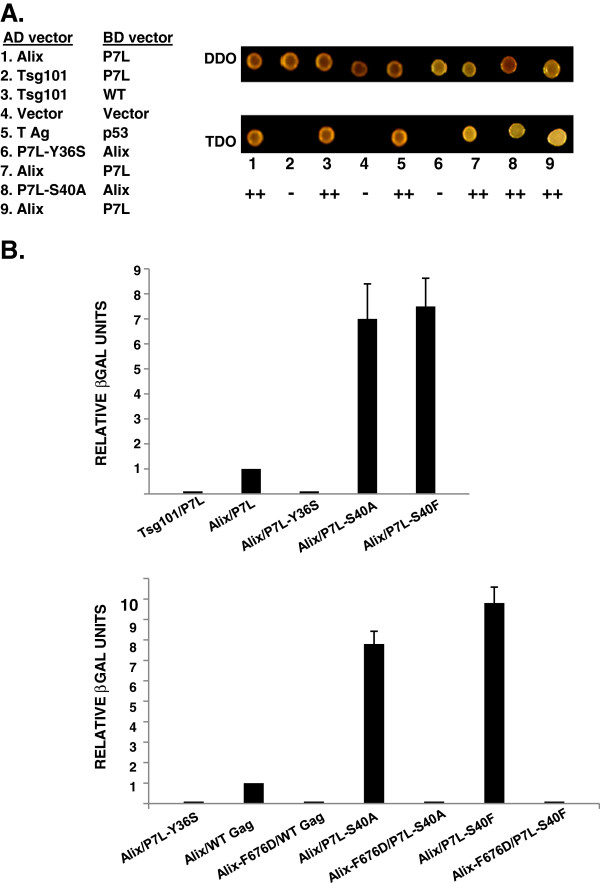
**Gag p6-S40 mutations increase binding to Alix in the yeast 2-hybrid assay.** Panel **A**, *Qualitative colony assay*. Co-transformed plasmids encoding protein pairs were tested for interaction under permissive and selective conditions using double drop-out media (DDO, Trp and Leu) and triple drop-out media (TDO, Trp, Leu, His), *respectively*. All transformed cells grew on the DDO media, indicating that plasmid pairs were expressed; only cells expressing interacting pairs grew on the TDO media. Panel **B**, *Quantitative liquid assay*. Beta-galactosidase assays were used to measure the relative strength of the S40 mutants plus Alix interaction. Beta-galactosidase units were normalized to P7L paired with Alix. Tsg101 plus P7L and Alix plus P7L-Y36S are negative controls. The S40A and S40F results represent five independent clones tested in 5 independent assays. Panel **C**, *The beta*-*galactosidase signal is undetectable when the S40 mutants are paired with Alix F676D*. WT Gag, P7L-S40A and P7L-S40F were each paired with wild type Alix or its F676D mutant which does not bind to Gag. Alix paired with Y36S serves as the negative control. Two independent clones were run in duplicate for each pairing. All values were normalized to WT paired with Alix.

The S40 residue is within the L_35_YPX2S_40_L motif that recruits Alix, but is not an obvious contributor to binding based on structural studies [29,30]. In contrast, the Y36 residue in the Alix-binding motif contacts Alix [28] and would therefore be expected to disrupt Gag-Alix interaction following substitution of Ser. To confirm this presumption, we tested the effect of S40 mutations on Gag-Alix interaction using the well-established yeast 2-hybrid assay. The yeast 2-hybrid assay (Y2H) for protein-protein interaction has been used in several previous studies to assess HIV-1 Gag-Alix interaction [11,13,41-43]. In particular, Lazert et al [43] examined the interaction of full-length Alix with the p6 minimal region (aa 31–46) that had been previously implicated in ALIX recognition and found that introducing Ala at residue 40 was well-tolerated. However, since Lazert et al also found that the interaction was modulated by regions outside of the binding site in both p6 and Alix proteins (*e.g*., the p6 aa 25–28 “hinge” and the Alix C-terminal Pro-rich domain), we examined the interaction between full-length Gag and full-length ALIX using a similar assay. Briefly, in this assay, proteins of interest that are fused to the DNA-binding (BD) or the activation domain (AD) of the yeast transcriptional activator, Gal 4, can interact and thereby promote expression of reporter genes [44]. Relative quantification of the protein/protein interaction strength was monitored by testing for growth on selection media or by measuring the β-galactosidase activity in yeast cells co-transformed with the BD- and AD- expressing plasmids. As shown in Figure 4, *panel A*, all of the AD- and BD-chimeric protein pairs tested grew on the double drop-out selection media (DDO, Trp and Leu), indicating that the plasmid pairs were expressed. When tested on the triple drop-out media (TDO, Trp, Leu and His), which tests for protein-protein interaction, the positive controls [*i.e*., full-length Alix paired with P7L-Gag, Tsg101 paired with WT Gag, and T Ag paired with p53 (*lanes 1*, *7 or 9*; *3 and 5*, *respectively*)] interacted, as expected. The negative controls (*i.e*., Tsg101 with P7L, empty vector paired with empty vector), and P7L-Y36S-Gag paired with Alix, gave no growth signal also as expected (*lanes 2*, *4*, *and 6*, *respectively*). Co-expression of P7L-S40A and Alix produced a signal that indicated their interaction (*lane* 8). *Panel B* shows quantification using the beta-galactosidase reporter. As expected, no signals were obtained for the negative control samples Tsg101 with P7L or P7L-Y36S paired with Alix. In contrast, the double mutant, P7L-S40A-Gag was observed to increase the binding to full-length Alix an average of 7-fold compared to P7L-Gag. This result was highly reproducible. In 5 independent trials, binding was stimulated 4-, 7-, 7-, 9-, and 10-fold (p < 0.05). Similar results were obtained for S40F. To ensure that the enhancing effect of the S40 mutation was directly attributable to Alix binding to the LYPX_2_SL motif in the p6 region, the inactivating mutation F_676_D [28] was introduced into the interacting V domain of Alix. As shown in *panel C*, this mutation reduced Alix binding to WT Gag, P7L-S40A and P7L-S40F to the level of interaction with the negative control, Y36S-Gag. We conclude that S40 mutation altered the binding of full-length Alix to full-length Gag and that the alteration is directly attributable to the interaction between the Alix V domain and the L domain-2 motif in p6.

### Disrupting Alix interaction with S40F restores spherical particle formation

**Figure 5:**
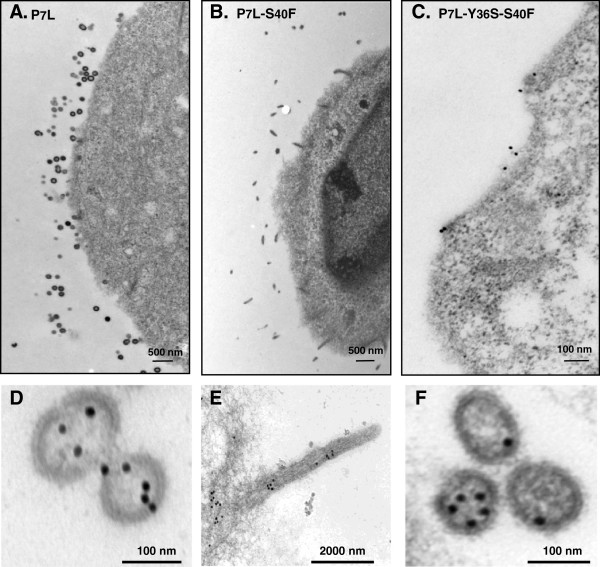
**Disrupting Alix interaction with S40F restores spherical particle formation.** COS-1 cells were transfected with plasmids expressing HA-tagged -P7L **(panels A and D)**, P7L-S40F **(panels B and E)**, or P7L-Y36S-S40F **(panels C and F)**. Panels **C-F**, cells were prepared for examination by immunoelectron microscopy as described in Methods. Bars in panels **A**, and **B** measure 500 nm; bars in panels **C**, **D** and **F** measure 100 nm; bar in panel **E** measures 2000 nm.

As described above, the yeast 2-hybrid assay revealed that the substitution of F or A for Ser40 in full-length P7L-Gag increased binding to the full-length Alix compared to the parental interaction. If the enhanced Alix binding is responsible for production of the filopodia-like membrane extensions, then disrupting Alix binding should reduce formation of the membrane extensions despite the negative impact on VLP release efficiency due to inhibition of L domain-2 function. To examine this possibility, we choose to block Gag-Alix interaction by introducing the Y_36_S mutation into the Gag-P_7_L-S_40_F construct. As described above, the Y_36_S mutation inhibited Alix binding to Gag (Figure 4) and also L domain-2-dependent release (see below). Examination by immunoelectron microscopy (Figure 5) showed that formation of the membrane extensions on cells transfected with DNA encoding P_7_L-S_40_F (*panels B and E*) was completely suppressed in cells expressing P_7_L-Y_36_S-S_40_F (*panels C and F*). *Panel C* shows that although gold-tagged P_7_L-Y_36_S-S_40_F accumulated at the cell periphery, the formation of filopodia-like structures was not induced. Additionally, formation of 100 nm spherical VLPs similar to those detected in the parental sample (*panels A and D*) was restored, although VLPs were detected at a much lower frequency in the P_7_L-Y_36_S-S_40_F sample compared to the parent. The results indicate that residue S40 plays a key role in budding and suggest that its effect on Alix binding was responsible for production of the filopodia-like membrane extensions containing Gag.

### The S40F mutation increases the disruption of virus release due to nonfunctional L domains-1 and −2

**Figure 6:**
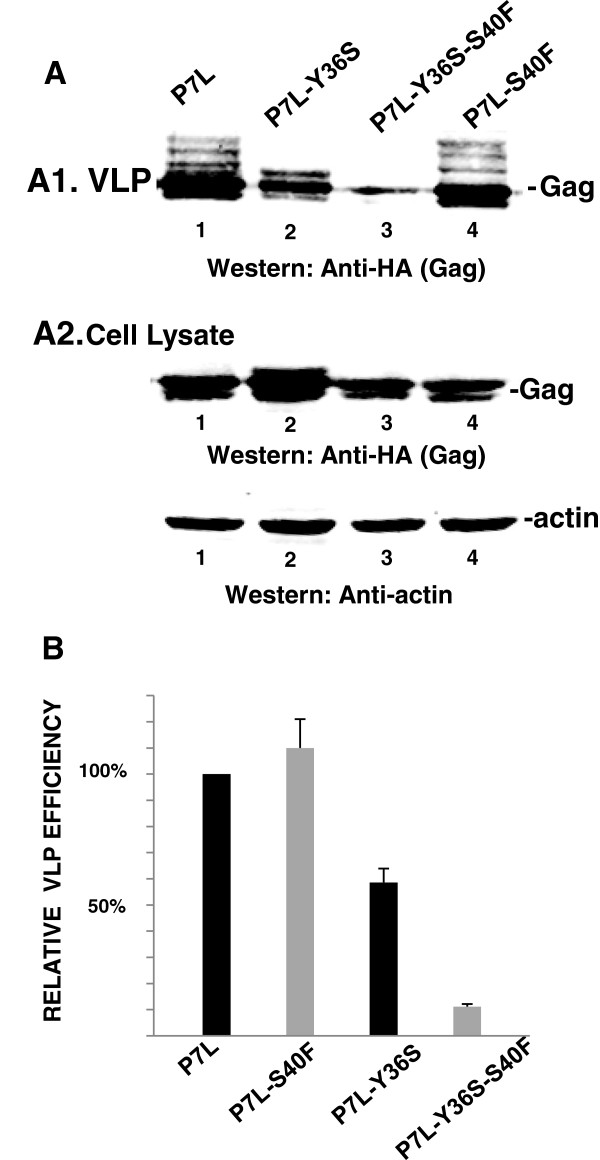
**The S40F mutation increases the disruption of virus release due to nonfunctional L domains-1 and −2.** Panel **A**, COS-1 cells transfected with DNA encoding the indicated constructs were analyzed by Western blotting after preparation of VLPs and cell lysates. Panel **B**, Quantitative analysis of VLP release efficiency [VLP/(VLP + Gag from cell lysate)] were determined as described in Methods (n = 7; 3 independent constructs of P_7_L-S_40_F-Gag).

The budding defects resulting from S40F mutation became increasingly more pronounced with L domain disruption, increasing in severity from reduced separation of the bud from the cell when the S_40_F mutation was introduced into L domain-2 (Figure 1B2) to reduced separation of buds from each other as well as from the cell when the S_40_F mutation was combined with L domain-1 disruption (Figures 2D; 3B2, 5E). Figure 6 shows the effect on budding when the S_40_F mutation was combined with disrupting mutations in both L domain-1 (P_7_L) and L domain-2 (Y_36_S) to produce P_7_L-Y_36_S-S_40_F. As noted above, under these conditions, budding is facilitated by certain isoforms in the Nedd4 family of ubiquitin E3 ligases working in conjunction with determinants in the C-terminal domain (CTD) of the capsid (CA) region in Gag [16,17]. As shown in *lane 3*, the introduction of the S_40_F mutation reduced virus production compared to the parental P_7_L-Gag (*lane 1*), P7_L_-Y_36_S-Gag (*lane 2*) or P_7_L-S_40_F-Gag (*lane 4*). Quantitative analysis indicated that VLP release efficiency was inhibited an average of 10-fold compared to P_7_L (n = 7 using 3 independent constructs of P_7_L-S_40_F-Gag; to ensure that this result was due to S40F, the entire Gag gene was sequenced). Thus, under these conditions, where both L domain-1 and −2 were disrupted, the budding defect resulting from the S40F mutation was even more pronounced, *i.e*., manifested in a significant block to viral particle release from cells.

### The S40F mutation partially rescues the block to budding imposed by mutations in the CA NTD

**Figure 7:**
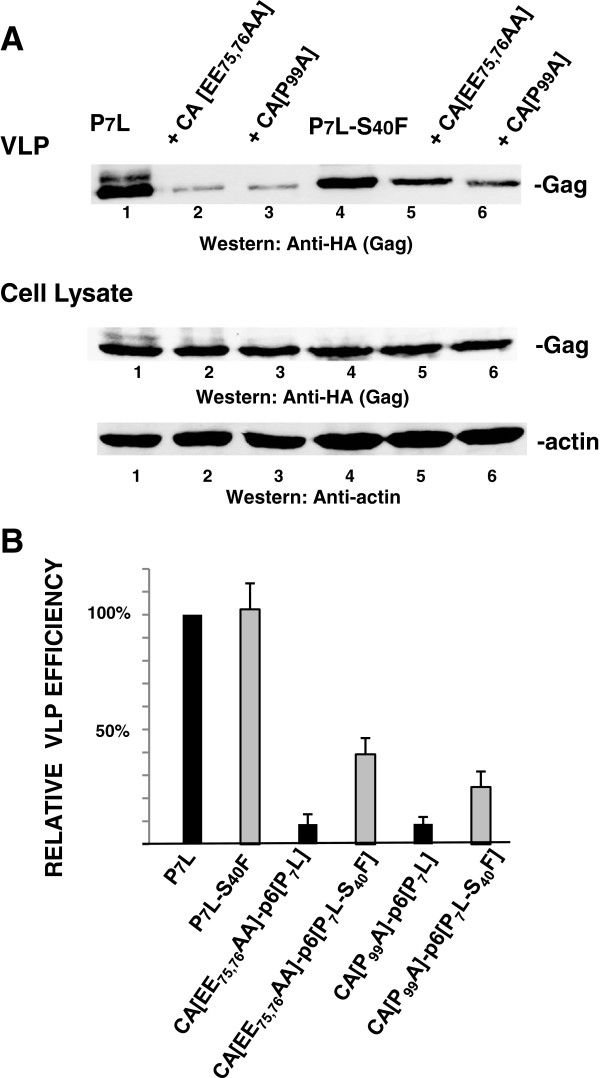
**The S40F mutation partially rescues the block to VLP release efficiency imposed by mutations in the CA NTD.** Panel **A**, COS-1 cells transfected with DNA encoding the indicated constructs were analyzed by Western blotting after preparation of VLPs and cell lysates. Panel **B**, Quantitative analysis of relative VLP release efficiency [VLP/(VLP + Gag from cell lysate)] were determined as described in Methods (n = 3).

**Figure 8:**
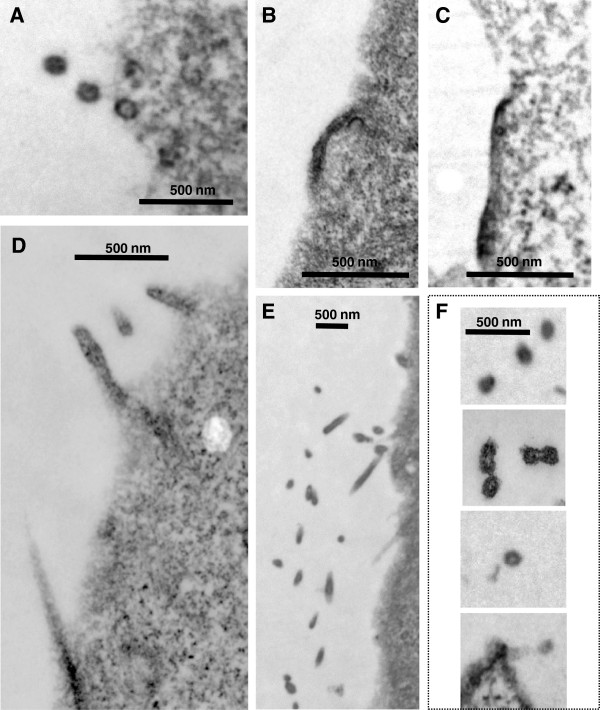
**The S40F mutation suppresses the block to budding imposed by CA[EE**_**75**_**,**_**76**_**AA] and CA[P**_**99**_**A].** The samples described in the legend to Figure 7 were analyzed by electron microscopy. **F. CA[P99A]-p6[P7L-S40F]** Panel **A**, P7L; panel **B**, CA [EE_75,76_AA]-p6[P_7_L]; panel **C**, CA[P_99_A]-p6[P_7_L]; panel **D**, P7L-S40F; panel **E**, CA[EE_75,76_AA]-p6[P_7_L-S_40_F]; and panel **F**, CA[P_99_A]-p6[P_7_L-S_40_F].

Our studies revealed that the effects of the P_7_L, Y_36_S and P_7_L-Y_36_S mutations, which all block budding at a late stage, were aggravated by the S_40_F alteration. Interestingly, residues in the N-terminal domain (NTD) of CA also contribute to budding since mutation of EE75,76 or P99 to Ala has been shown to permit Gag accumulation on the plasma membrane but block the subsequent membrane evagination, thereby blocking the budding process at an early stage [13,45,46]. To determine whether the S_40_F mutation impacts the phenotype resulting from the CA mutations, we constructed CA[EE_75,76_AA]-p6[P_7_L-S_40_F] and CA[P_99_A]-p6[P_7_L-S_40_F] and investigated VLP release efficiency by Western analysis. As shown in Figure 7, particle production by the parental P_7_L-Gag construct (*panel A*, *lane 1*) was reduced significantly by introduction of the CA mutations to form CA [EE_75,76_AA]-p6[P_7_L] (*lane 2*) and CA[P_99_A]-p6[P_7_L] (*lane 3*). Gag accumulation within the cell was not significantly affected but VLP release efficiency was inhibited ~10-fold, n = 3 (*panel B*). This outcome is expected, based on previous studies [13,45,46]. In contrast, ~3- to 4- fold more VLPs were detected in the media of cells expressing the mutants containing S_40_F (*lanes 5 and 6*) as compared to those lacking it (*lanes 2 and 3*). As Gag accumulation within the cell was not similarly increased, the increased VLP amounts reflected higher VLP release efficiency (*panel B*). This indicated that the S_40_F mutation impacted these mutants in a positive manner, in contrast to its effect on the p6 and CA CTD budding determinants. Supporting this, examination of the CA[EE_75,76_AA]-p6[P_7_L-S_40_F] (*panel E*) and the CA[P_99_A]-p6[P_7_L-S_40_F] (*panel F*) samples by electron microscopy (Figure 8) revealed VLPs similar in size and morphology to those detected in the parental P_7_L sample (*panel A*), albeit at lower frequencies (~10-30% of parental P_7_L), as well as elongated particles, similar to P7L-S40F. In contrast, the CA[EE_75,76_AA]-p6[P_7_L] and CA[P_99_A]-p6[P_7_L] samples revealed the expected dense staining underlying the plasma membrane and no VLPs (*panels B and C*). This indicated that the S_40_F mutation alleviated the defect imposed by the CA mutations, at least partially. The fact that similar results were obtained using two different CA mutants that exhibit the membrane curvature defect strongly suggests that the S_40_F mutation can complement their budding defect. Thus, the S_40_F mutation appeared to partially rescue the early budding defect imposed by mutation of the CA NTD determinants and to aggravate the block to late budding directed by the CA CTD and p6 determinants. These findings implicate the S40 residue in regulation of an important but previously unrecognized budding function.

## Discussion

Our study identifies a conserved residue in Gag p6 whose mutation had broad impact on budding: The S40F (and S40A, *not shown*) mutations (*i*) interfered with complete separation of particles from the plasma membrane in Tsg101-mediated budding; (*ii*) exacerbated the block to membrane scission in Alix-mediated budding; and (*iii*) inhibited bud release in Nedd4-facilitated egress. S40 mutation also attenuated the block to membrane evagination and scission imposed by mutations in the CA NTD, permitting more efficient VLP release to occur compared to the CA parents. Thus, S40 mutation affected the budding programs directed by all of the budding determinants identified to date in HIV-1 Gag. Studies to date indicate that HIV-1 release is most dependent on the L domain-1 PTAP-TSG101 Interaction. Nevertheless, Gag has conserved L domain-2 and determinants in the CA CTD domain that drive release when both L domain-1 and-2 are disrupted. The retention and conservation of these multiple late domains implies that they provide the virus with an evolutionary advantage, *e.g*., opportunities for synergistic interactions between different late domains in different cell types. The S40 polymorphisms A and F investigated here were revealed to us in HIV-1 variants that emerged in infected individuals, consistent with the notion that the mutations conferred an advantage. In this case, the advantage could be to virus spread since patients with these mutations exhibited high virus load and low CD4 counts that apparently were not controlled by ART or combo-ART. It was the dominance of variants with these mutations in the host quasi-species that prompted us to examine the possibility that the mutations were linked to the L domain function.

Our findings suggest that S40 is important in the context of the Gag precursor (in addition to any impact it may have in the context of the Gag-Pol precursor). An obvious choice for a Gag assembly function for which S40 is important involves Gag-Alix interaction as S40 resides within the L-domain 2 motif that serves as docking site for Alix. Alix has a modular structure that may permit it to function in multiple cellular pathways (reviewed in [47-49]). We found that mutations of S40 to F and A were accompanied by novel phenotypes, *i.e*., greater Alix binding (in the yeast 2-hybrid assay) and disruption or rescue of budding determinants. Thus, in combination with other determinants in Gag besides the ones we tested or other regions in the genome that can influence the budding process, a putative S40 regulatory function could provide multiple opportunities to influence viral budding modes and release efficiency and thus extend viral fitness.

Both S40F and S40A exhibited increased interaction with Alix in the yeast 2-hybrid assay and induced the formation of filopodia-like membrane extensions when transfected into mammalian cells. This suggested that the two phenotypes were linked, *i.e*., that the change in the Alix-Gag interaction might have resulted in formation of the unusual budding structures. We provided evidence to support this hypothesis by demonstrating that disrupting Alix interaction with the S40F mutant restored spherical particle formation while suppressing filopodia production. Residues 1 to 359 of Alix comprise the highly conserved Bro1 domain that binds the ESCRT-III component Chmp4 [50] and also actin [51]. Residues 360–716 of Alix comprise a second folded domain, designated as the “V” domain. The N-terminal “arm” of the V domain binds the actin-binding protein, cortactin [51]; the C-terminal “arm” of the domain binds viral LYPX_2_S_40_L [28,29]. Filopodia-like structures can result when cortactin is recruited by Alix to the cell periphery and there induces polymerization of cortical actin through Rho family GTPase signaling [52-56]. As Alix associates with both cortactin and Gag through the V domain, it is possible that the enhanced Gag-Alix binding resulting from the S40F mutation promotes formation of the filopodia-like structures in regions of the membrane where L domain-2-mediates VLP formation. Alternatively, the increased binding of Gag to the Alix V domain might have affected functions related to the Alix Bro domain, such as recruitment of CHMP4.

The dramatic effect of S40 mutation on the quality of the budding event was suppressed by preventing Alix-Gag interaction, clearly implicating Alix in the S40 function. Since we observed that S40 mutations altered Gag-Alix interaction in the yeast 2-hybrid assay, it is possible that the mutation interfered with Alix binding to its ESCRT or cytoskeletal protein partners as suggested above. However, as S40 mutation manifested the greatest impact on viral particle release efficiency when L domains-1 and −2 were both disrupted but also resulted in more subtle phenotypes under less disruptive conditions, we favor the view that mutation of this residue perturbs a previously unrecognized budding-related function. As noted above, when L domain-1 is disrupted, budding is directed by Alix, however, recruitment of a Nedd4 family member is also required [15]. Also as noted above, when L domain-1 is disrupted, Nedd4 family members can promote release, whether or not L domain-2 is intact [16,17] and siRNA-mediated depletion of endogenous Nedd4 prevents release [16]. It seems likely then that S40 mutation can prevent Nedd4 from contributing to budding directed by L domains −1 and −2. Conversely, the observation that S40 mutation interfered with budding mediated by determinants known to direct release through different cellular factors suggests that Nedd4 contributes to HIV budding even in conditions where its involvement has not yet been established, *i.e*., when directed by L domain-1 or CA NTD determinants. HIV-1 Gag lacks the conventional PY motif that is known to recognize Nedd4. However, we observed that its binding partner Tsg101 can associate with some family members [57] and also that Tsg101 can functionally replace Nedd4 function is some aspects of Avian Sarcoma Virus (ASV) Gag release [27]. Moreover, the motif in which S40 resides in Gag p6, LYPLXS_40_L is very similar to that found in another Alix binding partner, the membrane-associated protein MD 9/syntenin (LYPSL; [58,59]). In the case of syntenin, the motif, along with multimerization determinants in the protein, are essential for its interaction with ubiquitin. Also possibly pertinent to our findings, the overexpression of syntenin was observed to enhance formation of filopodia, linking its unusual interaction with ubiquitin to the formation of these structures. Intriguingly, the Ser residue in the motif is subject to phosphorylation and its modification regulates the interaction of syntenin with ubiquitin. These results define an unprecedented ubiquitin-dependent pathway regulated by a kinase. By analogy, the S40 residue in Gag p6 could become phosphorylated and serve as a platform for Nedd4-mediated ubiquitin modifications involved in budding. In this event, the substitution of any amino acid except possibly Thr would be expected to have deleterious effects. It has recently been suggested that the p6 region modulates the membrane interactions of HIV-1 Gag and that phosphorylation of S40 amplifies that interaction significantly [60].

It will be of interest to determine which of the afore-mentioned mechanisms underlies the impact of S40 mutation on L domain function observed in our study. In any case, the identification of a novel p6 role in viral assembly and budding may provide new targets and/or strategies for design of anti-viral agents.

## Conclusions

The substitution of Phe for a conserved residue in Gag p6, S40, can differentially affect L domain function. Our findings suggest that residue S40 is a determinant of a previously unrecognized role of p6 that impacts bud formation and release.

## Methods

### Plasmids and reagents

Plasmids pHIV-1-gag-HA tagged with hemagglutinin (HA) [61], pNL4-3ΔEnv [62] and pIIIB-Env3-1 [63] have been previously described. Yeast plasmid pGAD containing the full-length Alix (pGAD-Alix) was a generous gift from Dr. F. Bouamr (NIH). Yeast plasmid pGBT9 containing Gag was constructed by subcloning codon-optimized Gag [64]. Mutations in Gag and Alix were constructed by site-directed mutagenesis (Agilent). Gag-related proteins were detected using a polyclonal antibody against the native form of the capsid protein [65] or by a mouse monoclonal (Sigma-Aldrich) to the HA tag. Actin levels were detected by using a monoclonal antibody against actin (Sigma-Aldrich).

### Viral particle infectivity

COS1 cells were co-transfected with pNL4-3ΔEnv (wild type or constructs) and pIIIB env3-1 plasmids. At 48 h post-transfection, the culture medium was passed through a 0.45um pore size filter to separate virus from cells. The concentration of p24 in the filtered medium was determined by ELISA (Immunodiagnostics, Inc.) and equivalent amounts of p24 were used to infect MAGI cells [39]. Efficiency of infectivity at 48 hrs was measured as described [66]. As a control to verify that equivalent amounts of Env was expressed in each transfection, virus preps were run on SDS-PAGE followed by Western blot analysis using anti-HIV envelope antibodies (Immunodiagnostics, Inc.).

### Yeast 2-hybrid assays

HIV-1 Gag wild-type and mutants were tested for protein-protein interactions using the Matchmaker GAL4 Yeast Two Hybrid system (Clontech) with *Saccharomyces cerevisiae* strains AH109 (selective media assay) and Y190 (beta-galactosidase assay) as instructed by the manufacturer.

### Transfection and western blots

COS-1 cells were cultured in Dulbecco’s modified Eagle’s medium supplemented with fetal bovine serum (5%) and antibiotics (1%) to approximately 60% confluency at 37°C. The cells were transfected using XtremeGene reagent (Roche). At 48 h post-transfection, the culture medium was separated from the cells and the cells were washed in PBS and lysed in buffer (50 mM Tris, pH 7.4, 137 mM NaCl, 1.5 mM MgCl_2_, 1 mM EDTA, 1% Triton X-100, 10% glycerol) containing protease inhibitors (Roche). Virus particles were passed through a 0.45um pore size filter and isolated by ultracentrifugation through a cushion of 20% sucrose at 36,000 rpm for 90 min at 4°C using a Beckman SW41 rotor. Proteins were separated on 10 or 12% SDS polyacrylamide gels and identified by Western blotting. Proteins were visualized using an infrared-based imaging system (Odyssey, LI-COR Biotechnology). The secondary antibodies used to detect protein expression were Alexa Fluor 680 goat anti-mouse IgG (Molecular Probes, 1:10,000) and IRDyeTM800-conjugated affinity purified goat anti-rabbit IgG (Rockland, 1:10,000). For analysis of virus-like particle (VLP) release efficiency, measurements of bands corresponding to Gag in VLPs and cell lysates were made using the Li-Cor Odyssey software version 2.1.15. Release efficiency was defined as the ratio of the signal intensity value for the VLP-associated Gag to the sum of the values for VLP-associated Gag plus cell lysate-associated Gag [VLP/(VLP + Gag from cell lysate)].

### Electron microscopy

Cells grown on ACLAR film were fixed in 4% paraformaldehyde/0.1% EM grade glutaraldehyde in PBS, soaked in 2% osmium tetroxide, dehydrated in a graded series of ethyl alcohol solutions and embedded in Durpan resin. Eighty nm ultrathin sections were counterstained with uranyl acetate and lead citrate and viewed with a FEI Tecanal BioTwinG2 electron microscope. Immunogold labeling was as described in Ehrlich et al [67].

## Abbreviations

ESCRT: Endosomal sorting complex required for transport; CTD: C-terminal domain; NTD: N-terminal domain; PR: Protease; VLP: Virus-like particle.

## Competing interests

The authors declare that they have no competing interests.

## Authors’ contributions

SW, MC, MK, KSK, BW, BS, CC, LE, MP, KA, and HB were involved in data acquisition; SW, BW, CC, HB, LE and CAC participated in data analysis and interpretation; SW, LE and CAC designed the studies and wrote the manuscript; all authors read drafts and approved the final manuscript.
